# Functions of Insulin-like Peptide Genes (*CsILP1* and *CsILP2*) in Female Reproduction of the Predatory Ladybird *Coccinella septempunctata* (Coleoptera: Coccinellidae)

**DOI:** 10.3390/insects15120981

**Published:** 2024-12-11

**Authors:** Shanshan Feng, Da Wang, Qiuju Qin, Ke Chen, Wenjing Zhang, Yunzhuan He

**Affiliations:** College of Plant Protection, Hebei Agricultural University, Baoding 071000, China; shanshanet@163.com (S.F.); qiujuqin@163.com (Q.Q.); ck1270121229@sina.com (K.C.); zhang091288@sina.com (W.Z.)

**Keywords:** insulin-like peptides, insulin pathway, female reproduction, *Coccinella septempunctata*

## Abstract

Insulin-like peptides (ILPs) are important peptide hormones in insects, particularly involved in regulating physiological processes such as growth, development, and reproduction. However, the specific roles of ILPs in the reproduction of natural enemy insects remain unknown. In this study, two ILP genes, *CsILP1* and *CsILP2*, were cloned and their functions were analyzed in female *Coccinella septempunctata* L. (Coleoptera: Coccinellidae). The open reading frames (ORFs) of *CsILP1* and *CsILP2* were 384 bp and 357 bp, respectively. The expression of *CsILP1* increased on the 6th day after eclosion, reaching its peak on the 12th day, while *CsILP2* levels showed a significant increase on the 6th day and then stabilized. In different tissues, *CsILP1* was highly expressed in ovaries, while *CsILP2* predominated in elytra. Injection of dsRNA targeting *CsILP1* and *CsILP2* resulted in the down-regulation of insulin pathway genes. The relative expression of ovarian development-related genes *Vasa*, *G2/M,* and *Vg* was reduced by 82.50%, 89.55%. and 96.98% in *dsCsILP1*-treated females, and by 42.55%, 91.36%, and 55.63% in *dsCsILP2*-treated females. Furthermore, substantial decreases in 14-day fecundity were observed, with reductions of 89.99% for *dsCsILP1* and 83.45% for *dsCsILP2*. These results confirm the regulatory functions of *CsILP1* and *CsILP2* in female *C. septempunctata* reproduction.

## Simple Summary

Insulin-like peptides are known to be crucial endocrine hormones that influence various physiological processes, including growth and reproduction in insects. However, the specific roles of insulin-like peptides in the reproduction of natural enemy insects remain to be known. *Coccinella septempunctata* is an effective biological control agent and it is of great biocontrol significance to study the functions of insulin-like peptide genes in female reproduction of this natural predator. In this study, we cloned two insulin-like peptide genes and analyzed their functions in female *C. septempunctata*. It was found that silencing these insulin-like peptide genes resulted in significant down-regulation of ovarian development-related genes, leading to a prolonged pre-oviposition period, decreased fecundity, and reduced hatching rates of female *C. septempunctata*. These findings confirm the regulatory functions of these insulin-like peptide genes in female *C. septempunctata* reproduction and enhance our understanding of peptide hormones in natural enemy insects, contributing to improved biological pest control strategies.

## 1. Introduction

Insulin-like peptides (ILPs) are bioactive polypeptides in insects that act as endocrine hormones, playing a critical role in maintaining physiological homeostasis [1,2,3,4]. For insects, ILP was first identified in *Bombyx mori*, and it was designated as bombyxin [5,6,7]. Subsequently, more than 30 ILPs were identified in *B. mori* [8,9,10]. Up to now, eight ILPs have been found in *Drosophila melanogaster* [2,3], seven in *Anopheles gambiae*, five in *Anopheles stephensi* [11], eleven in *Acyrthosiphon pisum* [12], and five in *Leptinotarsa decemlineata* [13]. Different ILPs show different levels of expression in various tissues [4,10]. For instance, in adult *D. melanogaster*, ILP2 was most highly expressed in the brain, while ILP3 was in the midgut muscle, ILP6 was in the fat body, ILP7 was in the abdominal neuromeres and ILP8 was in the ovary [4,14]. In *Aedes aegypti*, the *ILPs* 1, 3, 4, 7, and 8 were specifically expressed in the brain, *ILP2* in the ovary, *ILP5* in the carcass, and *ILP6* in the fat body [15]. In *B. mori*, bombyxins A-G showed high expression in the brain [10], bombyxin-Y in the fat body and ovary [9,16,17], bombyxin-Z was highly expressed in follicular cells, and bombyxin-X exhibited the highest expression in the fat body [9]. This tissue-specific expression observed among ILPs contributes to the functional diversity in the regulation of growth, development, metabolism, and reproduction [2,10,18].

ILPs regulate insect reproduction mainly through the insulin pathway [19,20,21,22,23]. In the insulin pathway, ILPs act as upstream regulatory factors, binding to the insulin receptor (InR) and phosphorylating it. The phosphorylated InR further binds to and phosphorylates the insulin receptor substrate (IRS). The phosphorylated IRS transmits the signal downstream via phosphoinositide 3-kinase (Pi3k) [19]. Pi3k consists of a regulatory subunit (Pi3k-R) and a catalytic subunit (Pi3k-C). Phosphorylated IRS binds to Pi3k-R, activating Pi3k-C, which in turn phosphorylates phosphatidylinositol-4,5-bisphosphate (PIP2) to phosphatidylinositol-3,4,5-triphosphate (PIP3). When the concentration of PIP3 accumulates to a certain level, it activates downstream protein kinases AKT [22]. Activated AKT cascades downstream effector proteins to regulate cell growth, development, and differentiation [24]. Among the downstream effector proteins related to reproduction, protein G2/mitotic-specific cyclin-B (G2/M) is a cell-cycle regulatory protein that mediates the cells from interphase to division. Since vigorous reproduction in insects is associated with active cell division, G2/M is regarded as an indicator of reproductive system development [25]. Protein VASA is a member of the DEAD-box family, and it functions in regulating cell proliferation. VASA is widely expressed in insect germ cells, and required for oogenesis [26]. Vitellogenin (Vg) is a glucose–lipid complex protein with high molecular weight [27]. The synthesis of protein Vg in the fat body determines the vitellogenesis [27,28]. Therefore, the expression levels of Vasa, Vg, and G2/M are often used to assess ovarian development at the molecular level [25,26,27,28].

*Coccinella septempunctata* L. (Coleoptera: Coccinellidae) is a natural predator of many pests such as aphids, spider mites, and scale insects [29,30]. It is characterized by strong reproduction, long lifespan, and wide distribution [31,32]. As an effective biological control agent, its reproductive capacity has been heavily researched [33,34]. Insect reproduction is regulated by various endocrine hormones [27,35,36,37], especially juvenile hormone (JH) and peptide hormones [38]. JH has been proven to be necessary for female ovarian maturation [37,38,39], while the role of peptide hormones like ILPs remains unclear. In this study, two ILP genes (named *CsILP1* and *CsILP2*) were cloned from *C. septempunctata*, and their expression profiles and functions in female reproduction were verified. These results will enhance our understanding of the molecular mechanisms by which peptide hormones regulate the reproduction of natural enemy insects, and promote biological control of pests.

## 2. Materials and Methods

### 2.1. Insects

Seven-spot ladybird beetles (*C. septempunctata*) were captured from wheat fields (38°82′ N, 115°45′ E) at Hebei Agriculture University, Baoding, Hebei, China. Subsequent generations of larvae and adults were reared under conditions of (24 ± 1) °C and (60 ± 5)% relative humidity with a 16:8 h light/dark photoperiod, and fed on *Megoura japonica* Matsumura. Larvae were reared singly in 4 cm diameter Petri dishes. Newly emerged adults were paired, and each couple was transferred into a 180 mL clear plastic cup.

*M. japonica* was reared for generations in the Insect Physiology and Toxicology Laboratory of Hebei Agricultural University under conditions of (23 ± 1) °C and (50 ± 5)% relative humidity. They were fed on fresh pea seedlings [40].

### 2.2. RNA Isolation and cDNA Cloning of CsILP1 and CsILP2

Total RNA was isolated from the whole body of a 10-day-old female adult with the Total RNA Extraction Kit (Tiangen, Beijing, China) following the instructions. RNA contamination and degradation were monitored on 0.8% agarose gel electrophoresis. The concentration of RNA was examined by ultra-micro spectrophotometer MD2000C (Biofuture, Beijing, China). RNA samples (>1 μg, 28S:18S ≥ 1.0, OD260/280 = 1.8–2.2, OD260/230 ≥ 2.0) were used to synthesize cDNA using RT mix with DNase All-in-One (Suzhou, US EVERBRIGHT, China).

From the unpublished transcriptome database of *C. septempunctata*, we identified two differently expressed insulin-like peptides, namely *CsILP1* and *CsILP2*. Primers for amplification of *CsILP1*-ORF and *CsILP2*-ORF were designed using Primer Premier 6.0 software (Table S1). The sequences of *CsILP1* and *CsILP2* were PCR-amplified following protocol: 95 °C 3 min, (95 °C 15 s, 55 °C 15 s, 72 °C 3 min) *35 cycles, 72 °C 5 min, 4 °C hold, using 2× High-fidelity PCR Master Mix (Sangon, Shanghai, China). The PCR products with the expected size were cut from the gels, cloned into a *pEasy*-Blunt Zero Cloning vector (Transgen, Beijing, China), transferred into *Trans1*-T1 phage Resistant Chemically Competent cells, and then sequenced.

### 2.3. Bioinformatics Analysis

The ORFs of *CsILP1* or *CsILP2* were determined by NCBI Open Reading Frame Finder (https://www.ncbi.nlm.nih.gov/orffinder, accessed on 25 January 2024). The RNA sequences were translated to amino acid sequences by the Expasy Translate tool (https://web.expasy.org/translate/, accessed on 21 April 2024). Conserved Domains were searched by NCBI Conserved Domains Search (https://www.ncbi.nlm.nih.gov/Structure/cdd/wrpsb.cgi, accessed on 21 April 2024). The physical and chemical parameters were given by the Expasy ProtParam tool (https://web.expasy.org/protparam/, accessed on 21 April 2024). The presence of signal peptides was predicted by PredictProtein (https://predictprotein.org/, accessed on 16 May 2024). The amino acid sequences of ILPs in various insect species were searched from the NCBI database. The genomic contexts were predicted by NCBI Blast (https://blast.ncbi.nlm.nih.gov/Blast.cgi, accessed on 23 May 2024). DNAMAN 8.0 and MEGA7.0 software were used for sequence alignment. The Swiss Model (https://swissmodel.expasy.org/, accessed on 23 May 2024) was used for protein tertiary structure prediction. The ESPript 3.x (https://espript.ibcp.fr/ESPript/cgi-bin/ESPript.cgi, accessed on 23 May 2024) was used to edit sequence alignment results.

### 2.4. Expression Profiling Analysis of CsILP1 and CsILP2

To determine the expression profiles of *CsILP1* and *CsILP2* on different days after female eclosion, female adults were collected randomly on days 2, 4, 6, 8, 10, 12, and 14 after eclosion randomly. For each time point, 1 individual was treated as 1 replicate, and 4 biological replicates were set (a total of 28 individuals were used). To study the expression profiles of *CsILP1* and *CsILP2* in different tissues, we dissected the head, muscle, fat body, elytra, gut, and ovary of 10-day-old female adults. Each sample weighed 20 mg (each dissected from 20 females), with 4 biological replicates for each tissue.

Total RNA isolation and cDNA synthesis of each sample was performed as described above, and the cDNA concentration was diluted to 120–150 ng/μL for RT-qPCR. The primers for RT-qPCR were designed by Primer Premier 6.0, and *16S ribosomal RNA* (*16S rRNA*) and *β-actin* were selected as reference genes [41,42]. RT-qPCR was performed using 2*SYBR Green qPCR Master Mix (Suzhou, UElandy, China) on a Bio-Rad CFX machine (Bio-Rad, Hercules, CA, USA). The reaction volumes contained 10 μL of 2*SYBR Green qPCR Master Mix, 8 μL of ddH2O, 0.5 μL of each primer (10 μm), and 1 μL of cDNAs, and followed protocol: 95 °C for 2 min, followed by 40 cycles of 95 °C for 10 s, 58 °C for 15 s, and 72 °C for 15 s, then 95 °C for 1 min, 50 °C for 1 min, and 65 °C for 5 s. Technical replicates were performed 3 times.

### 2.5. RNA Interference Experiment

RNA Interference (RNAi) was applied to study the function of *CsILP1* and *CsILP2* in regulating the reproduction of female *C. septempunctata*, and *dsGFP* (green fluorescent protein) was used as the control [23]. The primers of dsRNA templates (*dsCsILP1*, *dsCsILP2,* and *dsGFP*, Table S2) were designed using Primer Premier 6.0 including a T7 promoter sequence. The dsRNA was synthesized according to the instructions of the T7 RiboMAX™ Express RNAi System kit (Promega, Shanghai, China), and then the dsRNA concentration was diluted to 1000 ng/μL. Afterward, 0.5 μL of dsRNA was injected into each abdomen of 4-day-old female adults (1:1 paired with male adults) using the 5 μL microinjector (Hamilton, Shanghai, China). For each treatment, 30 females were injected in 1 replication, from which 3 individuals were collected at 1, 3, and 5 days after injection to test the silencing effects of target genes, and 3 biological replicates were conducted. To further test the specificity of dsRNA, 3 individuals were collected at 3 days after injection. The relative expression levels of *CsILP2* after injection of *dsCsILP1* and levels of *CsILP1* after injection of *dsCsILP2* were measured and 3 biological replicates were conducted (a total of 270 female individuals were injected and 81 individuals were used to test the silencing effects and specificity of dsRNA at random).

In addition, to determine the function of *CsILP1* and *CsILP2*, female adults were selected 3 days after interference. The expressions of downstream genes in the insulin pathway *InR*, *IRS*, *Pi3k-R*, *Pi3k-C,* and *AKT* were tested. The expressions of genes *Vasa*, *Vg,* and *G2/M*, reflecting the developmental status of the female reproductive system, were also measured. The primers for RT-qPCR were designed by Primer Premier 6.0 (Table S3), and *16S rRNA* and *β-actin* were selected as reference genes [41,42]. For each treatment, 3 samples were set for 1 replicate and this was repeated 3 times (a total of 27 individuals were tested).

Then, 3 days after interference, female adults were dissected to observe the ovary status, 3 females were used for each treatment, and this was repeated 3 times (a total of 27 females were dissected). After interference, the pre-oviposition period, 14-day fecundity, and the color of eggs laid in the first spawning were recorded, 8 couples were set for 1 replicate and repeated 3 times [40,43]. For each treatment, the hatching rate was recorded, with 100 eggs collected in 1 sample, 4 samples for 1 replicate, and repeated 3 times.

### 2.6. Statistical Analysis

Relative expression of genes was calculated by 2^−∆∆Ct^ (Ct value is the number of qPCR cycles). IBM SPSS Statistics V21.0 was used for statistical analysis. For the RNAi experiment, the interference efficiency and specificity of dsRNA were analyzed by Student’s *t*-test. Other data were analyzed using one-way analysis of variance (ANOVA), with multiple comparisons conducted using Tukey’s HSD (*p* < 0.05). All data were shown as mean ± standard error (SE).

## 3. Results

### 3.1. Sequence Analysis of CsILP1 and CsILP2

The cDNA sequences of *CsILP1* and *CsILP2* were cloned and submitted to GenBank with accession Nos. OR656512 and OR656513, respectively. Both gene *CsILP1* and *CsILP2* were located on chromosome 3 (NC_058191.1), gene *CsILP1* has three exons and gene *CsILP2* has two exons.

**Figure 1:**
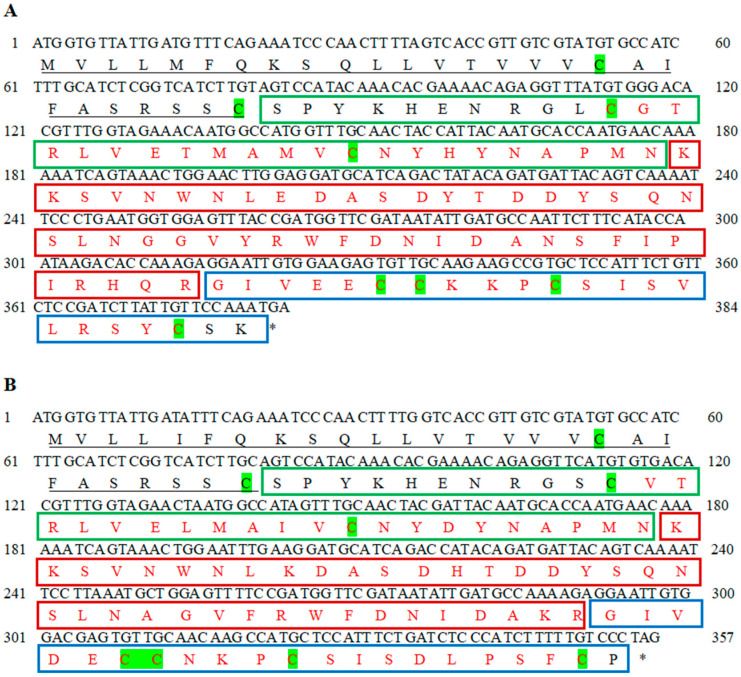
Gene sequences of *CsILP1* (**A**) and *CsILP2* (**B**) and the corresponding protein sequence from *C. septempunctata*. Signal peptides were “—” underlined, B chains were framed by green squares, C peptides were framed by red squares, A chains were framed by blue squares. An asterisk indicates the stop codon. Conserved domains were in red font. The characteristic cysteines of insulin-like sequences were highlighted in green.

The ORF of *CsILP1* was 384 bases, encoding a protein of 127 amino acids with a predicted molecular weight (MW) of 14.51 kDa and a theoretical isoelectric point (pI) of 8.34. The ORF of CsILP2 was 357 bases, and it encoded a protein of 118 amino acids with MW of 13.28 kDa and a pI of 6.68. The predicted CsILP1 and CsILP2 protein sequences both contained a Sec/SPI signal peptide (1-27aa) and a conserved IlGF_like superfamily domain (pfam00049), including B-chain, linker (C-peptide), and A-chain. For CsILP1, the domain spans 37-125aa, and for CsILP2, it spans 38-117aa (Figure 1).

**Figure 2:**
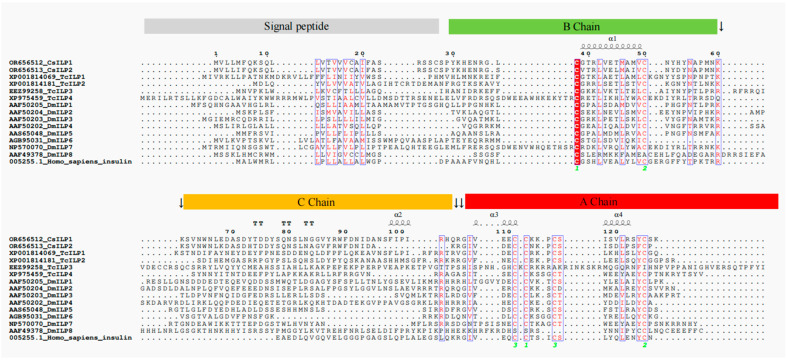
Multiple sequence alignment of CsILP1 and CsILP2 from female *C. septempunctata* with other insulin superfamily proteins. Sequences TcILP1, TcILP2b, TcILP3, and TcILP4 were derived from *T. castaneum*. Sequences DmILP1, DmILP2b, DmILP3, DmILP4, DmILP5, DmILP6, DmILP7, and DmILP8 were derived from *D. melanogaster*. The sequence name was preceded by GenBank accession number. Sequence Homo sapiens insulin was derived from humans. The canonical cleavage sites of the C-peptide are marked by black arrows. The common sequence is highlighted in red font. The green numbers represent the sites of the disulfide bonds, and the same numbers indicate they will be connected together after the cleavage of the C peptide.

Multiple sequence alignment (Figure 2) showed that CsILP1 and CsILP2 were highly conserved, with 79.53% identity. For CsILP1 and CsILP2, the two cysteine residues (-C-) in the B chain and the four cysteine residues (-C-) in the A chain are highly conserved across all proteins, including four sequences derived from *Tribolium castaneum* (TcILP1, TcILP2b, TcILP3, and TcILP4), eight sequences derived from *D. melanogaster* (DmILP1, DmILP2b, DmILP3, DmILP4, DmILP5, DmILP6, DmILP7, and DmILP8), and one sequence derived from humans (Homo sapiens insulin). At the C-terminal end of the B chain of CsILP1 and CsILP2, there are typical C-peptide cleavage sites occupied by two consecutive lysines (-K-K-). However, at the N-terminus of the A chain, CsILP2 has a typical C-peptide cleavage site composed of two consecutive arginine and/or lysine residues (-K-R-). In CsILP1, the lysine (K) at this position is replaced by glutamine (Q) (-Q-R-), and the -K-K- site appears at the eighth and ninth amino acid residues following this.

### 3.2. Expression Profiling of CsILP1 and CsILP2

**Figure 3:**
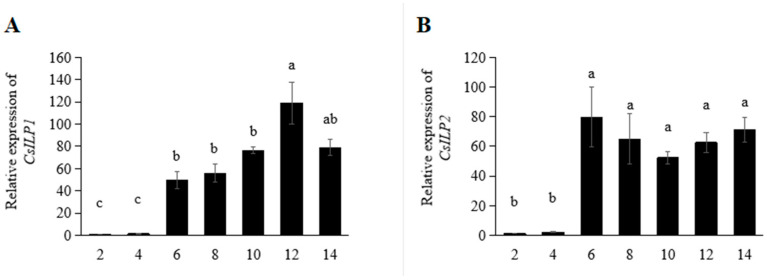
Mean (±SE) expression levels of *CsILP1* (**A**) and *CsILP2* (**B**) in *C. septempunctata* at different days (2, 4, 6, 8, 10, 12, and 14 days after eclosion). Different letters represented significant differences (ANOVA, Tukey’s HSD, *α* = 0.05).

The expression profiles of genes *CsILP1* and *CsILP2* at different days after eclosion are shown in Figure 3. For *CsILP1*, the expression level increased sharply on the 6th day, remained stable from the 6th to the 10th day, and peaked on the 12th day (*F* = 23.671; *df* = 6, 27; *p* < 0.001). The relative expression of the *CsILP2* gene significantly increased on the 6th day after eclosion and then remained stable (*F* = 9.013; *df* = 6, 27; *p* < 0.001).

**Figure 4:**
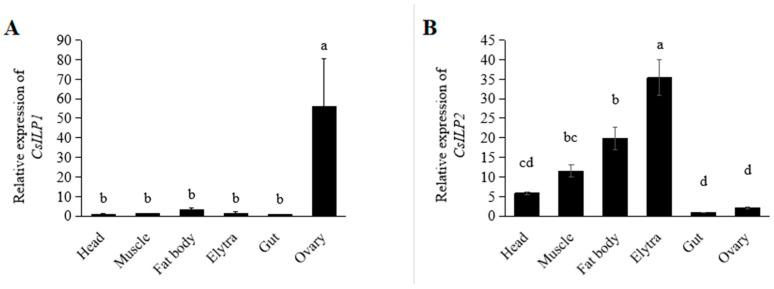
Mean (±SE) expression levels of *CsILP1* (**A**) and *CsILP2* (**B**) in different tissues of *C. septempunctata*. Different letters indicated significant differences (ANOVA, Tukey’s HSD, *α* = 0.05).

Tissue-specific expression profiles are shown in Figure 4. Both *CsILP1* and *CsILP2* were expressed in all tissues. *CsILP1* had the highest transcription levels in the ovary (*F* = 5.046; *df* = 5, 23; *p* = 0.005). *CsILP2* was the highest expressed in the elytra, followed by the fat body, and the lowest in the gut and ovary (*F* = 33.570; *df* = 5, 23; *p* < 0.001).

### 3.3. Functional Analysis of CsILP1 and CsILP2 by RNAi

**Figure 5:**
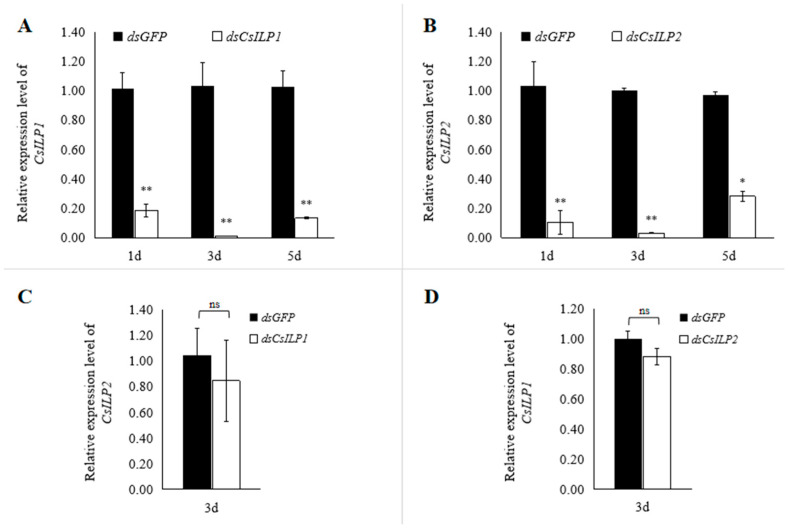
Silencing efficiency of *CsILP1* (**A**) and *CsILP2* (**B**) after dsRNA Interference in *C. septempunctata* and the specificity of dsRNA (**C**,**D**). Bars represented the mean ± SE, asterisk and “ns” indicate significant differences (Student’s *t*-test, * *p* < 0.05, ** *p* < 0.01).

To investigate the function of *CsILP1* and *CsILP2* in the reproductive process, females were injected with *dsCsILP1* or *dsCsILP2*, with *dsGFP* used as a control. The silencing efficiencies of *CsILP1* and *CsILP2* were analyzed by RT-qPCR after dsRNA injection. Results showed that, compared with the dsGFP control, the relative expression of *CsILP1* decreased by 82.02%, 99.06%, and 87.16% on days 1, 3, and 5 after *dsCsILP1* injection, respectively (day 1, *t* = 6.283, *df* = 4, *p* = 0.003; day 3, *t* = 5.695, *df* = 4, *p* = 0.005; day 5, *t* = 7.381, *df* = 4, *p* = 0.002, Figure 5A). Similarly, the relative expression of *CsILP2* decreased by 87.91%, 96.75%, and 78.67% on days 1, 3, and 5 after *dsCsILP2* injection, respectively (day 1, *t* = 14.426, *df* = 4, *p* < 0.001; day 3, *t* = 8.166, *df* = 4, *p* = 0.001; day 5, *t* = 4.088, *df* = 4, *p* = 0.015, Figure 5B). Compared to *dsGFP* control, there was no significant difference in the relative expression of *CsILP2* after *dsCsILP1* interference (*t* = 0.539, *df* = 4, *p* = 0.618), and vice versa (*t* = 1.710, *df* = 4, *p* = 0.162).

**Figure 6:**
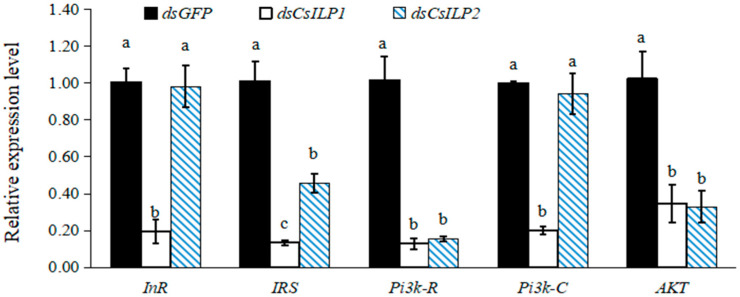
Mean (±SE) relative expression levels of insulin pathway genes in *C. septempunctata* 3 days after treatment with *dsGFP* control, *dsCsILP1* and *dsCsILP2*. Different letters represented significant differences in the same gene (ANOVA, Tukey’s HSD, *α* = 0.05).

Silencing *CsILP1* and *CsILP2* affected the expression of insulin pathway genes (Figure 6). Compared with the *dsGFP* control, the relative expression levels of downstream genes *InR*, *IRS*, *Pi3k-R*, *Pi3k-C,* and *AKT* decreased significantly by 80.95%, 87.77%, 88.58%, 79.96%, and 67.57%, respectively, on the third day post-*dsCsILP1* injection. For *dsCsILP2* injection, the relative expression levels of *InR* and *Pi3k-C* showed no significant difference, while *IRS*, *Pi3k-R,* and *AKT* expression decreased significantly by 55.45%, 85.97%, and 69.50%, respectively. Notably, the relative expression level of *IRS* in the *dsCsILP1* group was significantly lower than that in the *dsCsILP2* group, and there was no significant difference in the relative expression levels of *Pi3k-R* and *AKT* between the *dsCsILP1* and *dsCsILP2* groups (*InR*, *F* = 28.626, *df* = 2, 8, *p* = 0.001; *IRS*, *F* = 42.947, *df* = 2, 8, *p* = 0.000; *Pi3k-R*, *F* = 42.217, *df* = 2, 8, *p* = 0.000; *Pi3k-C*, *F* = 48.054, *df* = 2, 8, *p* = 0.000; *AKT*, *F* = 11.832, *df* = 2, 8, *p* = 0.008).

**Figure 7:**
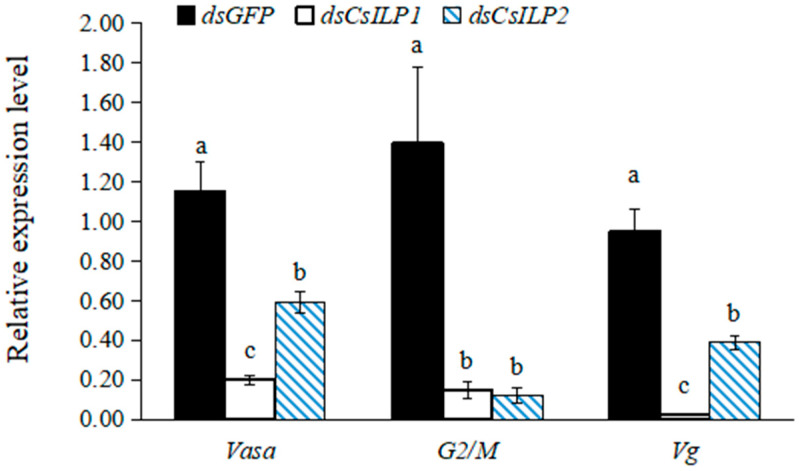
Mean (±SE) relative expression levels of reproduction-related genes in *C. septempunctata* after 3 days of treatment with *dsGFP* control, *dsCsILP1* and *dsCsILP2*. Different letters represented significant differences in the same gene (ANOVA, Tukey’s HSD, *α* = 0.05).

Expression of reproductive-related genes after *CsILP1* and *CsILP2* silencing was shown in Figure 7. Compared with *dsGFP* control, the expressions of *Vasa*, *G2/M*, and *Vg* decreased significantly by 82.50%, 89.55%, and 96.98%, respectively, on the third day after *dsCsILP1* injection. In the *dsCsILP2* injection group, the expression of *Vasa*, *G2/M*, and *Vg* decreased significantly by 42.55%, 91.36%, and 55.63%, respectively. Furthermore, the expressions of *Vasa* and *Vg* in *dsCsILP1* treatment were significantly lower than those in *dsCsILP2* treatment (*Vasa*, *F* = 21.337, *df* = 2, 8, *p* = 0.002; *G2/M*, *F* = 7.890, *df* = 2, 8, *p* = 0.021; *Vg*, *F* = 36.101, *df* = 2, 8, *p* < 0.001).

**Figure 8:**
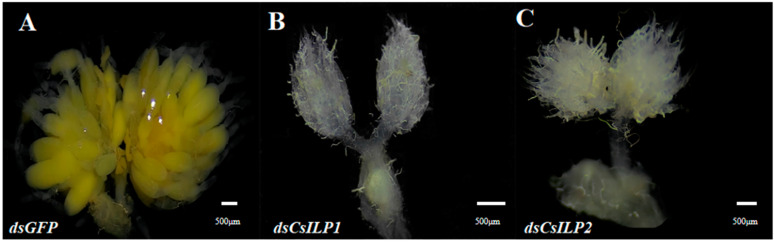
The ovarian development of female *C. septempunctata* 3 days after treatment with *dsGFP* control (**A**); *dsCsILP1* (**B**) and *dsCsILP2* treatment (**C**).

The ovarian development of female adults of *C. septempunctata* after *CsILP1* and *CsILP2* silencing is shown in Figure 8. Three days post-injection, the ovaries in the *dsGFP* control matured with yolk deposition (Figure 8A). The ovaries in the *dsCsILP1* (Figure 8B) and *dsCsILP2* (Figure 8C) treatment groups remained immature, with no yolk deposition observed.

**Figure 9:**
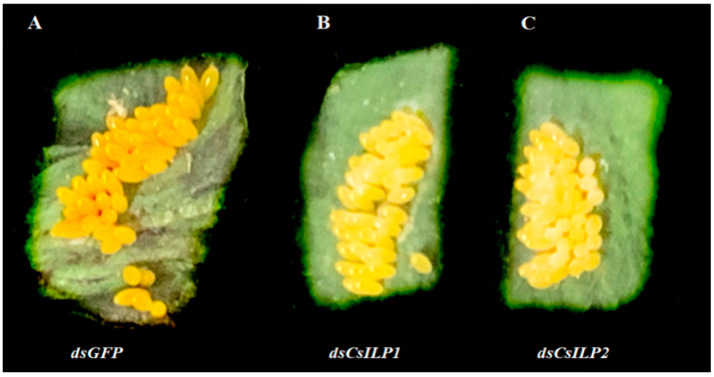
Eggs laid in the first spawning by female in *C. septempunctata* in *dsGFP* control group (**A**); *dsCsILP1* (**B**) and *dsCsILP2* treatment (**C**).

The effects of *CsILP1* and *CsILP2* silencing on the color of eggs are shown in Figure 9. The color of eggs laid in the first spawning by female adults *C. septempunctata* in *dsCsILP1* (Figure 9B) or *dsCsILP2* (Figure 9C) treatment group appeared notably lighter than that in the *dsGFP* control group (Figure 9A).

The effects of *CsILP1* and *CsILP2* silencing on the pre-oviposition, 14-day fecundity, and hatching rate are shown in Table 1. Compared with the *dsGFP* control, the pre-oviposition of female adults in the *dsCsILP1* and *dsCsILP2* treatment groups were significantly prolonged by 5.58 days and 4.54 days, respectively (*F* = 94.297; *df* = 2, 71; *p* = 0.048). The total 14-day fecundity was significantly lower in the *dsCsILP1* and *dsCsILP2* treatment groups, reduced by 89.99% and 83.45%, respectively (*F* = 88.020; *df* = 2, 71; *p* < 0.001), with no significant difference between the *dsCsILP1* and *dsCsILP2* treatment groups. The hatching rate for the *dsCsILP1* (83.11%) and *dsCsILP2* (82.44%) treatment groups was significantly lower than that of the *dsGFP* control group (90.11%), with no significant difference between the two treatment groups (Table 1; *F* = 15.239, *df* = 2, 35, *p* < 0.001).

## 4. Discussion

Insect ILPs belong to the insulin superfamily proteins (ISPs), which include insulin, insulin-like growth factor (IGF), relaxin, ILPs, etc. [44]. These ISPs are structurally conserved across divergent taxa, with the ILP gene encoding a propeptide that includes a signal peptide, a B-chain, a C-peptide linker, and an A-chain. Following the cleavage of the C-peptide, the B- and A-chains, linked by three disulfide bonds (formed by six cysteine residues), convert into an activated form [44]. In this study, two ILP genes, *CsILP1* and *CsILP2*, were cloned from *C. septempunctata*. The predicted protein sequences for both *CsILP1* and *CsILP2* contain a signal peptide and a conservative domain of IlGF_like superfamily (including a B-chain, a C-peptide, and an A-chain).

Multiple sequence alignment showed the cysteine residue sites are conserved across all sequences. CsILP2 has two typical C-peptide cleavage sites at the C-terminus of the B-chain and the N-terminus of the A-chain, each occupied by pairs of consecutive arginine and/or lysine residues (-K-K- and -K-R-). However, at the N-terminus of the A chain in CsILP1, the lysine (K) is replaced by glutamine (Q) (-Q-R-). The cleavage of the C-peptide is crucial for the structure and function of ISPs, and any mutation at the cleavage site may affect protein function [44]. Therefore, mutations in the cleavage site of c-peptide in CsILP1 might have some influence on its function.

On different days after female eclosion, the expression of *CsILP1* sharply increased on the 6th day and reached its peak on the 12th day, while the expression of *CsILP2* significantly increased on the 6th day and then remained stable. Previous research found that female *C. septempunctata* deposited yolk on the 6th to 8th day after eclosion, and reached the spawning peak around the 12th day. This consistency suggests that the expression dynamics of *CsILP1* and *CsILP2* may be closely related to the reproductive dynamics of *C. septempunctata* [39].

In most insects, ILPs are expressed in a variety of tissues, including the ovary, gut, fat body, brain, hemolymph, and carcass [4,12,45]. This diversity in tissue-specific expression often suggests functional differences among them. For instance, in *B. mori*, bombyxins A-G, secreted by brain neurosecretory cells, regulate nutrient-dependent growth, and metabolism, while the high expression of Bombyx IGF-like peptide (BIGFLP) in the fat body shows its function on ovarian development [4,10,17,46]. In *D. melanogaster*, *DmILP8*, which is highly expressed in the ovary, showed a clear reproductive relevance, and it was proposed to be a relaxin-like peptide and have gonadotropic functions [14,47,48,49,50]. Furthermore, the expression levels of ILPs are influenced by various environmental factors including temperature, circadian rhythm [51], pesticides [52], and nutritional conditions [48]. Many hormones and growth factors present in the elytra can alter body color and size in response to environmental changes, which in turn affects the growth, development, and reproduction of insects [53,54]. Therefore, we speculate that the expression of ILPs in the elytra may be related to environmental response. In this research, *CsILP1* was highly expressed in the ovaries and *CsILP2* in the elytra. These tissue-specific expressions of *CsILP1* and *CsILP2* indicate their relevance to reproduction and development, highlighting the importance of further investigating their mechanisms of action.

The functions of ILPs in regulating the female reproduction are common in many insects, such as *D. melanogaster* [14], *A. aegypti* [55], *T. castaneum* [56], *Spodoptera litura* [57], *Chrysopa septempunctata* [38], *Propylea japonica* [23], *Colaphellus bowringi* [58], *Chrysopa pallens* [36], *Chilo suppressalis* [59], and *Adelphocoris suturalis* [20]. In this study, females treated with *dsCsILP1* and *dsCsILP2* showed prolonged preoviposition periods, reduced 14-day fecundity, decreased hatching rates, and delayed ovarian development. At the molecular level, the expressions of reproduction-related genes *Vasa*, *Vg,* and *G2/M* were down-regulated in both treatment groups. These observations indicated that *CsILP1* and *CsILP2* have a regulatory role in the reproductive process of *C. septempunctata*. The reproductive development of insects involves oogenesis and vitellogenesis [60]. Gene *Vasa* is essential for oogenesis, *Vg* for vitellogenesis, and *G2/M* for the germ cell cycle [25,26,27,28,61]. Currently, research on female insect reproductive development mainly focuses on vitellogenesis [58,62], with less emphasis on oogenesis and the germ cell cycle. Investigating the differential regulatory effects of ILPs on oogenesis and vitellogenesis in female *C. septempunctata* will deepen our understanding of the regulatory mechanisms underlying insect ovarian development.

ILPs regulate the reproduction of insects primarily through insulin pathways [4,17,20]. In this study, silencing *CsILP1* led to significant downregulation of downstream genes *InR*, *IRS*, *Pi3k-R*, *Pi3k-C*, and *AKT*. Silencing *CsILP2* resulted in significant downregulation of *IRS*, *Pi3k-R*, and *AKT* genes, while *InR* and *Pi3k-C* were not significantly affected. This suggests that *CsILP1* and *CsILP2* differentially affect the mRNA expression level of genes in the insulin pathway, and their specific mode of action in female reproduction remains to be further investigated. In the insulin pathway, the specific binding of ILPs and InR is a key transmembrane signal. InR is a receptor tyrosine kinase. The structures and functions of InRs vary in insects [60,63,64,65]. In *N. lugens* (Stål), two types of InRs, InR1 and InR2, have been found to play opposite roles in ovarian development regulation [65]. InR1 and InR2 have also been found in *T. castaneum* regulating reproductive development through different mechanisms [64]. In *D. melanogaster*, a leucine-rich repeat G protein-coupled receptor (LGR), a homolog of relaxin receptor, mediates the regulation of *DmILP8* in female reproduction [14,47,49]. Therefore, further explorations to clarify the interaction between different ILPs and different receptors are expected to deepen our understanding of the insulin pathway. Furthermore, genes in the insulin pathway can also regulate insect reproduction by interacting with other hormone pathways or nutrition pathways, such as the target of rapamycin (TOR) nutritional pathway and JH pathway [66,67,68,69]. Further exploration of the interaction mechanisms among the insulin pathway, JH pathway, and TOR pathway in regulating insect reproduction is of great significance for elucidating the endocrine regulation of insect reproduction.

In this study, *CsILP1* and *CsILP2* were cloned from *C. septempunctata*, and their expression patterns and roles in ovarian development and fecundity were investigated. These results clarified the role of *CsILP1* and *CsILP2* in female *C. septempunctata* reproduction, providing a basis for further studies on the molecular mechanisms involved. In the wild, *C. septempunctata* obtain nutrition from various small insects. Therefore, a deeper investigation into the interactions between the insulin pathway, TOR nutrient pathway and the JH pathway in reproductive regulation is of significant scientific importance for enhancing the application efficiency of releasing *C. septempunctata* in fields.

## Figures and Tables

**Table 1:** Effects of *CsILP1* and *CsILP2* silencing on reproduction of female adults *C. septempunctata*.

Treatments	Pre-Oviposition (Days)	14 Days Fecundity (Eggs per Female)	Hatching Rate (%)
*dsGFP*	10.17 ± 0.29 c	296.79 ± 22.54 a	90.11 ± 0.93 a
*dsCsILP1*	15.75 ± 0.36 a	29.71 ± 8.83 b	83.11 ± 1.23 b
*dsCsILP2*	14.71 ± 0.26 b	49.13 ± 13.03 b	82.44 ± 1.50 b

Note: data were mean ± SE; different letters indicated significant differences between treatments (ANOVA, Tukey’s HSD, *α* = 0.05).

## Data Availability

The raw data of the cDNA sequences of *CsILP1* and *CsILP2* were submitted to GenBank with accession Nos. OR656512 and OR656513.

## Supplementary Materials

The following supporting information can be downloaded at: https://www.mdpi.com/article/10.3390/insects15120981/s1. Table S1: the primers for cDNA Cloning of *CsILP1* and *CsILP2*. Table S2: the primers for RNA Interference. Table S3: the primers for RT-qPCR.
