# Supplementary material for: Functions of Insulin-like Peptide Genes (CsILP1 and CsILP2) in Female Reproduction of the Predatory Ladybird Coccinella septempunctata (Coleoptera: Coccinellidae)
Source: Insects. 2024 Dec 11;15(12):981. doi: 10.3390/insects15120981 (PMC11677109; doi:10.3390/insects15120981)
Supplement: Supplementary file 1 [file insects-15-00981-s001.zip › insects-3301928-supplementary.pdf]

Table S1. The primers for cDNA Cloning of *CsILP1* and *CsILP2*

Gene	Primer	Primer sequence (5' to 3')	Length
CsILP1	Forward	ATGGTGTTATTGATGTTTCAGAAATCC	27
	Reverse	TCATTTGGAACAATAAGATCGGAGAA	26
CsILP2	Forward	ATTGAGAAGATTCACATCCTCCAA	24
	Reverse	GTATCTACATCAGTAGCTAGGGACA	25

Table S2. The primers for RNA Interference

Gene	Primer	Primer sequence (5' to 3')	Length
dsGFP	Forward 1	taatacgactcactatagg GCCACAAGTTCAGCGTGTCCG	40
	Reverse 1	AGTTCACCTTGATGCCGTTCT	21
	Forward 2	taatacgactcactataggAGTTCACCTTGATGCCGTTCT	40
	Reverse 2	GCCACAAGTTCAGCGTGTCCG	21
dsCsILP1	Forward 1	taatacgactcactataggGCCATCTTTGCATCTCGGTCAT	41
	Reverse 1	CGGTAAACTCCACCATTCAGGG	22
	Forward 2	taatacgactcactataggCGGTAAACTCCACCATTCAGGG	41
	Reverse 2	GCCATCTTTGCATCTCGGTCAT	22
dsCsILP2	Forward 1	taatacgactcactataggTGCATCTCGGTCATCTTGTAGT	41
	Reverse 1	ACACTCGTCCACAATTCCTCTT	22
	Forward 2	taatacgactcactataggACACTCGTCCACAATTCCTCTT	41
	Reverse 2	TGCATCTCGGTCATCTTGTAGT	22

Table S3. The primers for RT-qPCR

Gene	Primer	Primer sequence (5' to 3')	Length
16S ribosomal RNA	Forward	GGACCTGCCCACTGAATTATTA	22
	Reverse	TTCTCATCAAACCATTCATACAAGC	25
β-actin	Forward	GATTCGCCATCCAGGACATCTC	22
	Reverse	TCCTTGCTCAGCTTGTTGTAGTC	23
CsILP1	Forward	ACCGTTGTCGTATGTGCCATCT	22
	Reverse	CGGCTTCTTGCAACACTCTTCT	22
CsILP2	Forward	CCGTTGCCGTATGTGCCATC	20
	Reverse	GCAACACTCGTCCACAATTCCT	22
InR	Forward	GTCAGCCAATGTCCGTCCGATA	22
	Reverse	GTAGCAAGGTCCCTCGCACAAT	22
IRS	Forward	AATGTGAGTCTGGCGAAGGAAT	22
	Reverse	AGGTCTACCAATGGGCTTGAGT	22
Pi3k-R	Forward	GCCAACACGGACACCTTGAAGA	22
	Reverse	CGCCTCGTTCCTTAGACACAGT	22
Pi3k-C	Forward	CAGGTTCTTCACAGATCGGAGA	22
	Reverse	GACAACGCAAGAGCATTCACAT	22
AKT	Forward	AACGACTACGGCAGGGCTGTA	21
	Reverse	AGGACCGCCACCTAACCTCAAT	22
Vasa	Forward	TTGCCACGCCAGGAAGACT	19
	Reverse	CGCTACCAACGATGCCTACG	20
Vg	Forward	AGGATTCGTGGACTCTGTGGAA	22

	Reverse	TGGTTCTTCTGTGAGCGACTCT	22
G2/M	Forward	GCGATGACAAGGACCAAGAGG	21
	Reverse	TCAGGTTGTATTCGTCGGCTAC	22
